# Analysis of the Culturable Skin Microbiome of Horses from Southern Germany

**DOI:** 10.3390/microorganisms13030623

**Published:** 2025-03-08

**Authors:** Mahdis Matinpour, Nadine Zettner, Kristin Neumann, Lisa Bäumer, Andreas Burkovski

**Affiliations:** 1 Microbiology Division, Department of Biology, Faculty of Sciences, Friedrich-Alexander-Universität Erlangen-Nürnberg, Staudtstr. 5, 91058 Erlangen, Germany; mahdis.matinpour@fau.de (M.M.); nadine.zettner@fau.de (N.Z.); 2 Mymicrobiome GmbH, Nürnberger Str. 108e, 96050 Bamberg, Germany; neumann@microbiome-friendly.com

**Keywords:** *Bacillus cereus*, equine skin, horse skin microbiome, *Macrococcus carouselicus*, MALDI-ToF MS, microbial community

## Abstract

Horses have close interactions with humans and are important as working animals and livestock. In contrast to smaller companion animals like cats and dogs, there is only little information available about their skin microbiome. The objective of this study was to identify and characterize the culturable cutaneous microbiome of healthy horses. Samples were taken from 14 horses from Southern Germany which were randomly enrolled in this study. Matrix-assisted laser desorption/ionization time-of-flight mass spectrometry (MALDI-TOF MS) was used as a method to detect the culturable microorganisms of horse skin. The most abundant culturable species of horse skin identified in this study include *Bacillus cereus*, *Bacillus pumilus*, *Carnobacterium inhibens*, *Exiguobacterium sibiricum*, *Macrococcus carouselicus*, *Macrococcus goetzii* and *Staphylococcus equorum*. Analyses of the bacteria across different body regions indicated the specific preferences of species for certain skin areas. In addition, our data hinted to an influence of the age of the horses tested and an influence between the four stables studied.

## 1. Introduction

Horses, like dogs and cats, have close interactions with humans, e.g., as working animals or livestock, but research on their skin microbiome remains scarce in comparison to that of smaller domesticated animals such as cats and dogs [1,2,3,4,5,6,7,8,9,10,11]. One of the earliest studies focusing on wounded horses identified an unknown bacterium from the *Acidobacteria* phylum, which was also prevalent in the healthy control horses of the study [12]. The first notable study on the healthy equine cutaneous microbiome, which indicated seasonal variation in the equine skin microbiota, identified 38 phyla and 1665 genera. The most common phyla across all samples were *Proteobacteria*, *Firmicutes*, *Actinobacteria* and *Bacteroidetes*, but their abundance varied across different body sites. At the genus level, nine genera were consistently found across different sites: *Psychrobacter*, *Macrococcus*, *Pseudomonas*, *Acinetobacter*, *Planomicrobium*, *Arthrobacter*, *Carnobacterium*, *Desemzia* and *Corynebacterium*, and it was indicated that Gram-negative bacteria are prevalent in the equine skin microbiome [13]. In the latest study on healthy horses, *Proteobacteria* was again identified as the most abundant phylum across all sites. *Actinobacteria*, *Firmicutes*, *Deinococcota* and *Bacteroidota* were also present, with varying abundances depending on the body site. At the genus level, *Corynebacterium* was the most abundant, followed by *Deinococcus* and *Macrococcus*. Additionally, within this study, the genera *Pseudomonas*, *Psychrobacter*, *Acinetobacter*, *Desemzia* and *Carnobacterium* were identified [11].

In the two most recent studies on the cutaneous microbiome of healthy horses [11,13], it was concluded that the skin site is a primary factor influencing the composition of the skin microbiota. The studies differed in respect to the conclusion of whether the individual horse is a major factor in shaping the skin microbiome or not. An additional key difference was the presence of *Deinococcota*, aerobic Gram-positive rods known for their high resistance to environmental stresses, which were abundant on the back of the horses in one study [11], but not in the other [13]. 

The differences between studies on the cutaneous microbiome of horses highlight the significant lack of research in this area. Furthermore, none of the studies identified bacteria at the species level, which is a crucial gap. Additionally, the use of 16S rRNA sequencing as the primary method in the mentioned studies may contribute to differences in the results obtained, since this method relies on the analysis of DNA isolated from the samples and detects dead and alive organisms. Taken together, this underscores the need for studies that employ diverse and concurrent methodologies to provide a more comprehensive understanding of the equine skin microbiome.

## 2. Materials and Methods

### 2.1. Sample Collection

**Figure 1:**
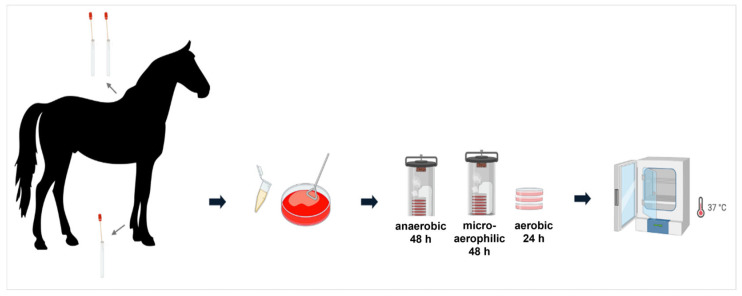
Sampling procedure. Samples were taken from the pastern and back of the horse using eSwab^®^-Liquid Amies Elution Swabs. Tubes containing the swabs were subsequently stored at 4 °C until further processing and samples were subsequently plated on Columbia blood agar plates and incubated at 37 °C under aerobic, microaerophilic and anaerobic conditions, respectively (created with BioRender; https://app.biorender.com; 6 February 2025).

eSwab^®^-Liquid Amies Elution Swabs (Copan, Brescia, Italy) were used to collect microorganisms from back and pastern (Figure 1) of 14 horses, which were randomly enrolled in this study from four different stables located in Southern Germany (Table 1). Sampling was carried out by the owners of the horses during routine skin care after careful training by and under the supervision of one of the authors (experienced microbiologists). To ensure sterility and minimize environmental contamination, particularly from the human microbiome, medical gloves and disinfectant spray were used during the sampling process. The hair at each site was parted to allow the swab to directly contact the skin. Each site was swabbed a minimum of five times using rotational and lateral movements with sterile swabs containing 1 mL of liquid Amies medium in specimen transport tubes. The tubes containing the swabs were subsequently stored at 4 °C until further processing and were transferred within 24 h to the laboratory where they were streaked out immediately. The participants were instructed carefully and gave written consent to participate in this study. According to the University’s ethical guidelines, approval was not required for this study.

### 2.2. Cultivation of Samples and Isolation of Pure Cultures

A total of 500 μL of phosphate-buffered saline (PBS; 137 mM NaCl, 2.7 mM KCl, 10 mM Na_2_HPO_4_ × 12 H_2_O, 2 mM KH_2_PO_4_, adjusted to pH 7.4 with phosphoric acid) was added to the tubes containing the swabs and Amies medium. Subsequently, 50 µL of this suspension from each tube was plated onto Columbia blood agar plates (Oxoid, Wesel, Germany). For each horse body site, the samples were cultured under three different conditions: aerobic, anaerobic and microaerophilic. Each condition was tested in triplicate on plates. The plates were incubated at 37 °C for 24 h under aerobic conditions, for 48 h under anaerobic conditions using a sample transport box with two Oxoid AnaeroGen sachets (Oxoid, Germany) and for 48 h under microaerophilic conditions also using a sample transport box with two Oxoid CampyGen sachets (Oxoid, Germany). For plates exhibiting excessive bacterial growth, 1:10 dilutions with PBS were performed to isolate individual colonies. From the diluted suspension, 50 µL was plated onto fresh blood agar plates. 

From the mixed cultures derived from the samples, bacterial colonies were carefully selected and labeled based on their morphological, color, size and hemolytic properties. Individual bacterial colonies were subsequently isolated from the agar plates using a sterile inoculation loop and transferred to fresh blood agar plates using the three-sector streaking method.

### 2.3. Identification of Bacteria

For optimal results with MALDI-TOF-MS, bacterial isolates from pure cultures were streaked on new blood agar plates and incubated at 37 °C under the required conditions (aerobic plates for 24 h, microaerophilic and anaerobic plates for 48 h). The plates were wrapped with Parafilm and shipped on the same day. The MALDI-ToF MS analysis was carried out by Ripac-Labor GmbH (Potsdam, Germany) and Labor Dr. Risch (Buchs, Switzerland).

In short, fresh bacterial colonies dissolved in formic acid, sterile water and ethanol were applied to polished or ground steel MALDI target plates and a matrix, typically consisting of α-cyano-4-hydroxycinnamic acid dissolved in 50% acetonitrile, and 2.5% trifluoroacetic acid was placed on top, and the plates were left to air dry at room temperature. As the solvent evaporated, the sample and matrix mixture co-crystallized, forming a solid deposit. The plate was loaded into the MALDI-TOF mass spectrometer. The MALDI process caused the sublimation and ionization of both the sample and matrix. Each spectrum was the sum of the ions obtained from 200 laser pulses taken in five distinct areas of the same spot. Using a TOF analyzer, these spectra were examined in the 1000–11,000 mass-to-charge ratio (m/z) range. The presence or absence of peaks within the generated spectacle serves as a fingerprint for a particular isolate, creating an MS profile. The software database is then used to compare and analyze these profiles [14,15,16].

## 3. Results

### 3.1. Identification of Culturable Bacteria by MALDI-ToF MS

A total of 146 bacterial species, species-like taxonomical units and species clusters from seven phyla and seventy-two genera were identified (Table 2). The most prevalent phyla across all samples were *Proteobacteria* (33 genera), *Firmicutes* (22 genera) and *Actinobacteria* (12 genera). The identified genera with the most members were *Bacillus*, *Pseudomonas*, *Macrococcus* and *Staphylococcus*.

### 3.2. Identification of Dominant Culturable Bacterial Species on Equine Skin

**Figure 2:**
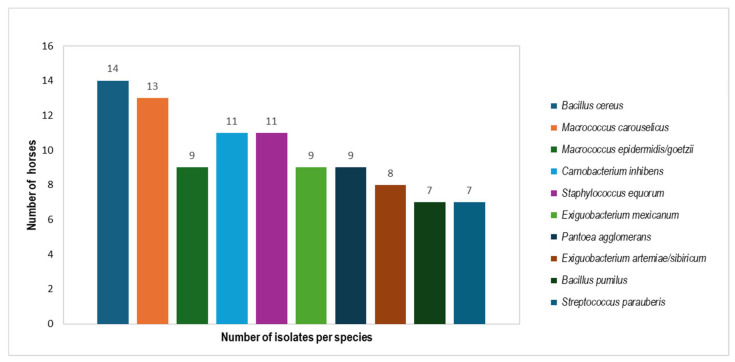
Overview of the dominant culturable bacterial species from equine skin samples. The bar chart provides a comprehensive visualization of the dominant bacterial species found in equine skin samples, independent of the anatomical body sites. The *X*-axis represents the different bacterial species, while the *Y*-axis shows the number of horses in which each bacterial species was found. Each bar is color-coded according to the bacterial species, with the count numbers displayed on top of each bar for clarity. The legend on the right provides the full names of the bacterial species corresponding to each color.

MALDI-TOF analysis of swab samples from the 14 horses tested revealed *B. cereus* as the most dominant species, being present on all horses. *M. carouselicus* was detected on 13 horses, while *C. inhibens* and *S. equorum* were found on 11 horses, respectively. *E mexicanum*, *M. epidermidis/goetzii* and *P. agglomerans* were present on nine horses. *E. artemiae/sibiricum* was found on eight horses. *S. parauberis* and *B. pumilus* appeared on seven horses (Figure 2).

*Bacillus cereus* is a Gram-positive, spore-forming, rod-shaped bacterium, isolated from a wide range of sources such as air, soil, water and food. *B. cereus* is a producer of cereulid and different enterotoxins, making it a widely distributed food-poisoning pathogen [17]. Due to the formation of highly resistant endospores, it is difficult to define a natural habitat for this species. In contrast to *B. cereus*, the closely related *Bacillus pumilus* is non-pathogenic. The species is found in water, sediments and soil and has plant growth promoting activity [18]. 

*Macrococcus* species are coagulase-negative, catalase- and oxidase-positive cocci. They belong to the *Staphylococcaceae* family and are closely related to the *Staphylococcus* genus. These bacteria are typically found as commensals in animals and are generally considered non-pathogenic in their hosts. However, limited information exists on the ecological distribution of *Macrococcus* among different mammalian species, with few studies documenting their presence on animal skin. *M. carouselicus* colonies grow to 5–7 mm in diameter on various media, appearing slightly convex, entire, buttery, glistening or opaque, and exhibiting a cream-to-light beige pigmentation. *M. goetzii* cells can be irregular or regular spherical cocci, depending on the medium. Their colonies have whole margins, are slightly convex, smooth, shiny, 1–2 mm in diameter and are non-pigmented [19]. 

The genus *Carnobacterium* has been isolated from cold, low nutrient environments, including Antarctic lake water, Arctic soil, pufferfish, permanently cold seawater, human faces and tomb mural paintings. They are known to tolerate high pressure environments, such as the vacuum packing process in food preservation. The use of lactic acid-producing bacteria like *Carnobacterium* spp. in food and bio-preservation is a growing area of research in the meat, dairy and seafood industries. Bacteriocins produced by these bacteria have antimicrobial properties that limit or inhibit the growth of foodborne pathogens. *C. inhibens* are catalase-negative and facultatively anaerobic, with small, gray colored, round colonies measuring 1–2 mm in diameter. Growth occurs within a pH range of 5.8–9.0 [20,21].

Staphylococci are part of the normal microbiota of the skin and mucous membranes of mammals and are also widely distributed in various environments, including soil, water and air, and various foodstuffs such as meat, cheese and raw milk. They are catalase-positive and salt-tolerant. *S. equorum* is coagulase-negative and is frequently detected in food processing environments and fermented foods, where it can inhibit the growth of undesirable microorganisms. *S. equorum* forms small colonies, and the subspecies *equorum* was originally isolated from healthy horses [22,23]. 

Members of the genus *Exiguobacterium* have been isolated from a wide variety of environments and are catalase- and oxidase-positive. *E. mexicanum* colonies are orange-, yellow- and cream-colored, with rods varying from 0.5 to 0.75 mm in diameter and 0.8–3.0 mm in length. *E. sibiricum* is adapted to cold environments, with mass mapping visualizing the differences in protein expression at various growth temperatures. The cells vary in shape and size depending on the temperature, being 0.8 mm long and 0.6 mm in diameter at 30 °C, but reaching up to 15 mm long at temperatures between 0 and 12 °C. The colonies are bright orange, convex, entire and shiny [24,25].

*P. agglomerans* is a mesophilic, Gram-negative, motile bacterium that was isolated from knee lacerations [26].

### 3.3. Major Bacterial Species at Different Anatomical Sites

**Figure 3:**
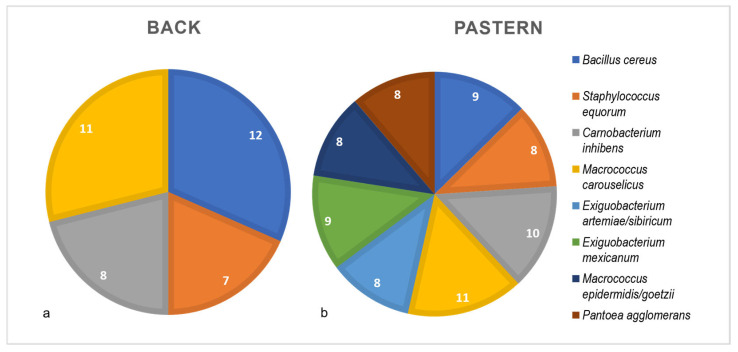
Distribution of major bacterial species at two anatomical sites on equine skin. Pie charts represent the prevalence of dominant bacterial species on the back (**a**) and pastern (**b**) of horses. Each color represents a specific bacterial species, with the number of horses harboring each species displayed inside its corresponding color-coded segment. The legend on the right provides the full names of the bacterial species corresponding to each color.

To identify the most prevalent species under varying skin conditions, their occurrence at specific body sites, namely the back and pastern, was analyzed. The analysis revealed that *B. cereus* was the most common species on the back, found on 12 individual horses, followed by *M. carouselicus* on 11 horses. Additionally, *C. inhibens* was identified on the back of eight horses, and *S. equorum* on seven horses (Figure 3a). In the pastern area, *M. carouselicus* was the dominant species, detected on 11 different horses. *C. inhibens* was identified on the pasterns of 10 horses. Moreover, *B. cereus* and *E. mexicanum* were found on nine out of fourteen horses. *E. artemiae/sibiricum*, *P. agglomerans*, *M. epidermidis/goetzii* and *S. equorum* were present on the backs of eight horses (Figure 3b).

### 3.4. Comparison of Dominant Culturable Bacterial Species in Different Stables

The analysis of horses from different stables (Table 3) revealed that *B. cereus* was present in all horses across all stables. Additionally, *M. carouselicus* and *C. inhibens* were common species in three out of the four stables. 

In stable A, a total of 28 different species were identified. Among these, *B. cereus*, *E. acetylicum*, *M. carouselicus* and *S. aureus* were common among the horses. Stable B had a total of 48 different bacterial species, with *B. cereus, C. inhibens, E. artemiae/sibiricum* and *M. carouselicus* being prevalent. Stable C exhibited the highest diversity, with a total of 92 different bacterial species. Common species in this stable included *B. cereus, B. pumilus, C. inhibens, E. mexicanum, M. carouselicus, P. agglomerans* and *S. parauberis*. In stable D, 41 species were identified, with *B. cereus, C. inhibens* and *S. equorum* being common in all horses.

### 3.5. Age-Dependency of Dominant Culturable Bacterial Species

**Figure 4:**
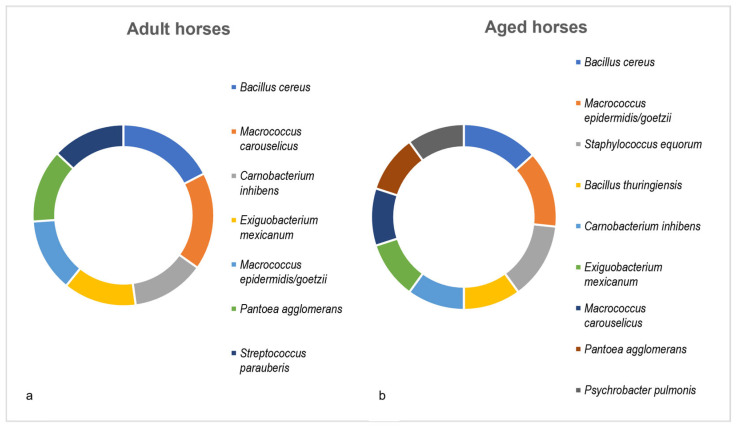
Dominant culturable bacterial species depending on age. (**a**) adult and (**b**) aged horses. Bacterial species presented were present in at least three out of four horses within each group.

For the age-related analysis, the horses were separated into two age groups (each with n = 4): adult horses between 2 and 9 years old and aged horses between 14 and 35 years old. The bacterial species had to be present in at least three out of four horses within each group. In the adult horses, a total of 99 different species were identified. *B. cereus* and *M. carouselicus* were found in all four horses. Other common bacterial species included *C. inhibens*, *E. mexicanum*, *M. epidermidis/goetzii*, *P. agglomerans* and *S. parauberis* (Figure 4a).

In aged horses, a total of 58 species were identified. *B. cereus*, *M. epidermidis/goetzii* and *S. equorum* were present in all horses. Additionally, *B. thuringiensis*, *C. inhibens*, *E. mexicanum*, *M. epidermidis/goetzii*, *P. agglomerans* and *P. pulmonis* were found in at least three out of four horses (Figure 4b).

## 4. Discussion

In this study, the most frequently identified genera in all samples were *Bacillus* (*Firmicutes*), *Pseudomonas* (*Proteobacteria*), *Macrococcus* (*Firmicutes*) and *Staphylococcus* (*Firmicutes*). Among these, the *Bacillus* genus demonstrated the highest species diversity. *B. cereus* was identified as the most abundant species in all horses, followed by *M. carouselicus*, *C. inhibens*, *S. equorum*, *E. mexicanum*, *M. goetzii*, *S. parauberis* and *B. pumilus*, all belonging to the *Firmicutes* phylum. In addition, *P. agglomerans*, a member of the Proteobacteria, and *E. sibiricum,* belonging to the Actinobacteria, were observed with a high frequency. In contrast, previous studies on the skin microbiome of healthy horses reported *Proteobacteria* and *Bacteroidota* to be the predominant phyla, with *Proteobacteria* being the most abundant one [11,13]. Notably, Strompfová and Štempelová [11] identified *Deinococcota* as one of the most abundant phyla, which was not found in a previous study [13] and was only less abundant here. Differences in results may be attributed to different geographical regions and the influence of different environmental factors. As shown previously, for example, high humidity and low temperature conditions were associated with a higher frequency of bacteria and variability in the skin microbiota and could be correlated with longitudinal and latitudinal variation in UV exposure (for a review, see [27]). As observed before [7,13], non-human mammals generally have a higher number of soil-associated taxa than humans. This difference is likely due to the fact that the animal’s skin, which is largely covered in dense fur, comes into more frequent contact with soil bacteria. This suggests a potential relationship between the skin microbiome of animals and the soil microbiome of their habitats. Additionally, the spores of certain species may be present in the fur of the studied horses, leading to their reactivation during cultivation in the lab.

The analysis of the dominant culturable bacterial species on the different body regions of horses, specifically the pastern and back, revealed similar bacterial populations, in general, but a greater diversity was observed on the pastern compared to the back. *B. cereus*, *M. carouselicus*, *C. inhibens* and *S. equorum* were identified as dominant species on both body sites, while *E. mexicanum*, *E. sibiricum*, *P. agglomerans* and *M. goetzii* were found only on the pastern. A lower diversity on the back was reported previously [11,13]. It was suggested that the reduced bacterial diversity on the back of horses might be due to factors such as the presence of sweat containing alkaline minerals, less exposure to the environment and frequent coverage by saddles. The higher diversity in the pastern area may be attributed to direct contact with soil and the horse’s environment, which influences the skin microbiome. However, it is important to note the need for more research and repeated studies to fully understand the differences in skin microbiome across anatomical sites in healthy horses. Furthermore, the microbiome of the pastern would be interesting in respect to its influence on equine pastern dermatitis [28,29,30,31].

The analysis of the equine skin microbiome in horses from different stables showed the common distribution of *B. cereus* in all four stables, and the presence of *C. inhibens* and *M. carouselicus* in three stables. These bacteria were identified as part of the dominant species on horse skin, making their detection across multiple stables plausible. In contrast, certain bacteria were specific to individual stables. This variation may be attributed to differences in hygiene and feeding routines, the population density of horses and the frequency of their interactions and their contact with humans and other animals, such as dogs, cats and goats within each stable. Research on the dog skin microbiome has shown that humans and dogs living together can impact each other’s skin microbiome [13]. Similarly, a sheep population’s bacterial communities are influenced by their handlers, with human biology and lifestyle playing significant roles [32]. This interaction could also apply to horses in different stables, potentially explaining the observed differences.

In addition, age-dependent influences were observed. Adult horses exhibited greater overall microbiome diversity, while older horses displayed more similar bacterial communities. This observation suggests that younger horses have more diverse microbiomes, whereas the microbiomes of older horses are more uniform. These findings align with those reporting a decrease and stabilization in the skin microbiota diversity with age in donkeys [33]. The uniformity observed in aged horses could be due to their less frequent riding routines, leading to reduced contact with humans and the environment. Additionally, it might be related to changes in the functionality and metabolism of the various microbiomes within the skin ecosystem, a hypothesis that warrants further comprehensive research to fully understand these differences, especially in light of the limitation of this study, the very low number of horses included and the rather broad age groups. Follow-up studies may include newborn or very young horses in addition to involving higher numbers of animals.

Despite these insights, the impact of age on the horse skin microbiome remains underexplored. The limited sample size in this study underscores the need for larger scale research to validate these findings and to provide a more comprehensive understanding of age-related changes in the equine skin microbiome. Future studies may include quantitative analyses of culturable microbiomes, approaches for the better detection of fastidious bacteria as well as yeasts and fungi.

## 5. Conclusions

The study presented here focused on the analysis of the culturable microbiome of healthy horses. The results obtained are complementary to recently published studies using 16S rRNA sequencing for the taxonomic identification of species, and provide further experimental evidence that species identified as dominant by sequencing methods are detectable by culturing as well.

## Figures and Tables

**Table 1:** Overview of horses acquired for this study. Abbreviations: m, male; f, female.

No.	Stable	Breed	Sex	Age	Sterilized	Routine Riding
1	A	Irish Tinker	f	3.5	no	yes
2	A	Irish Tinker	m	12	yes	yes
3	A	Irish Tinker	m	17	yes	yes
4	B	Speed Racking	m	1.5	no	no
5	B	Curley	m	7	yes	yes
6	B	Shetland Pony	m	10	yes	no
7	B	Connemara Pony	m	10	yes	yes
8	C	Andalusier	m	15	yes	yes
9	C	American Paint	m	8	yes	yes
10	C	Moritzburger	m	5	yes	yes
11	D	Bayerisches Warmblut	f	19	no	yes
12	D	Pinto	f	15	yes	yes
13	D	Quarter	m	31	no	no
14	D	Quarter	m	14	yes	yes

**Table 2:** Equine skin bacteria identified by MALDI-ToF MS.

Phylum	Genus	Species
*Firmicutes*	*Aerococcus*	*Aerococcus viridans*
	*Bacillus*	*Bacillus altitudinis*
		*Bacillus amyloliquefaciens*
		*Bacillus cereus*
		*Bacillus infantis*
		*Bacillus licheniformis*
		*Bacillus mycoides*
		*Bacillus pumilus*
		*Bacillus safensis*
		*Bacillus subtilis*
		*Bacillus subtilis/amyloliquefaciens*
		*Bacillus thuringiensis*
		*Bacillus xiamenensis*
	*Butyrivibrio*	*Butyrivibrio MabrTax32*
	*Carnobacterium*	*Carnobacterium gallinarum*
		*Carnobacterium inhibens*
		*Carnobacterium jeotgali*
		*Carnobacterium maltaromaticum*
		*Carnobacterium viridans*
	*Desemzia*	*Desemzia incerta*
	*Enterococcus*	*Enterococcus mundtii*
	*Lactococcus*	*Lactococcus raffinolactis*
	*Latilactobacillus*	*Latilactobacillus fuchuensis*
	*Lysinibacillus*	*Lysinibacillus* sp.
	*Macrococcus*	*Macrococcus bohemicus/epidermidis/goetzii*
		*Macrococcus bovicus/brunensis*
		*Macrococcus canis*
		*Macrococcus carouselicus*
		*Macrococcus caseolyticus*
		*Macrococcus epidermidis/goetzii*
		*Macrococcus equipercicus*
		*Macrococcus flavus*
		*Macrococcus hajekii*
		*Macrococcus* spp.
	*Mammaliicoccus*	*Mammaliicoccus vitulinus*
	*Micrococcus*	*Micrococcus* sp.
		*Micrococcus flavus*
		*Micrococcus luteus*
	*Neobacillus*	*Neobacillus bataviensis*
	*Niallia*	*Niallia circulans*
	*Paenibacillus*	*Paenibacillus amylolyticus*
		*Paenibacillus beijingensis*
		*Paenibacillus MabrTax5*
		*Paenibacillus illinoisensis*
		*Paenibacillus lautus*
		*Paenibacillus saccharophilum*
	*Paraclostridium*	*Paraclostridium benzoelyticum/bifermentans*
	*Peribacillus*	*Peribacillus simplex*
		*Peribacillus simplex/butanolivorans/muralis/frigoritolerans*
	*Priestia*	*Priestia aryabhattai/megaterium*
		*Priestia megaterium*
	*Psychrobacillus*	*Psychrobacillus lasiicapitis*
		*Psychrobacillus MabrTax3*
		*Psychrobacillus psychrodurans*
		*Psychrobacillus lasiicapitis*
	*Solibacillus*	*Solibacillus silvestris*
	*Staphylococcus*	*Staphylococcus aureus*
		*Staphylococcus chromogenes*
		*Staphylococcus delphini/intermedius/pseudintermedius*
		*Staphylococcus equorum*
		*Staphylococcus sciuri*
		*Staphylococcus succinus*
		*Staphylococcus vitulinus*
	*Streptococcus*	*Streptococcus equinus*
		*Streptococcus equorum*
		*Streptococcus gallolyticus*
		*Streptococcus parauberis*
*Proteobacteria*	*Acinetobacter*	*Acinebacter gandavensis*
		*Acinetobacter lwoffii*
		*Acinetobacter radioresistens*
		*Acinetobacter schindleri*
		*Acinetobacter terrestris*
		*Acinetobacter towneri*
	*Acetobacter*	*Acetobacter ascendens*
	*Aeromonas*	*Aeromonas aquatica/encheleia*
		*Aeromonas bestiarum/salmonicida*
		*Aeromonas encheleia*
		*Aeromonas media/rivipollensis*
		*Aeromonas* spp.
	*Alcaligenes*	*Alcaligenes faecalis*
	*Aliidiomarina*	*Aliidiomarina haloalkalitolerans*
	*Bowmanella*	*Bowmanella yangjiangensis*
	*Colwellia*	*Colwellia MabrTax21*
	*Dyella*	*Dyella amyloliquefaciens*
	*Erwinia*	*Erwinia billingiae*
	*Escherichia*	*Escherichia vulneris*
	*Haemophilus*	*Haemophilus parahaemolyticus*
	*Kosakonia*	*Kosakonia sacchari*
	*Leclercia*	*Leclercia adecarboxylata*
	*Lelliottia*	*Lelliottia amnigena*
	*Marinomonas*	*Marinomonas arctica/shanghaiensis*
	*Massilia*	*Massilia aurea*
	*Mycobacterium*	*Mycobacterium MabrTax33*
	*Neisseria*	*Neisseria musculi*
	*Pannonibacter*	*Pannonibacter indicus*
	*Pantoea*	*Pantoea agglomerans*
		*Pantoea* sp.
		*Pantoea vagans*
	*Petrocella*	*Petrocella atlantisensis*
	*Photorhabdus*	*Photorhabdus heterorhabditis*
	*Planococcus*	*Planococcus glaciei*
	*Pseudomonas*	*Pseudomonas alloputida/capeferrum/hunanensis*
		*Pseudomonas azotoformans/carnis/lactis/paralactis*
		*Pseudomonas frederiksbergensis*
		*Pseudomonas koreensis*
		*Pseudomonas lundensis*
		*Pseudomonas MabrTax108*
		*Pseudomonas MabrTax304*
		*Pseudomonas MabrTax342*
		*Pseudomonas proteolytica*
		*Pseudomonas* spp.
		*Pseudomonas tolaasii*
		*Pseudomonas umsongensis*
	*Psychrobacter*	*Psychrobacter pulmonis*
	*Rahnella*	*Rahnella aquatilis complex*
	*Rhizobium*	*Rhizobium MabrTax114*
	*Serratia*	*Serratia myotis/quinivorans*
	*Shewanella*	*Shewanella* sp.
	*Stenotrophomonas*	*Stenotrophomonas rhizophila*
	*Testudinibacter*	*Testudinibacter aquarius*
	*Thauera*	*Thauera sedimentorum*
	*Thioalkalivibrio*	*Thioalkalivibrio versutus*
*Actinobacteria*	*Agrococcus*	*Agrococcus citreus*
	*Arthrobacter*	*Arthrobacter citreus*
	*Corynebacterium*	*Corynebacterium gallinarum*
		*Corynebacterium kalinowskii*
	*Curtobacterium*	*Curtobacterium flaccumfaciens*
	*Exiguobacterium*	*Exiguobacterium acetylicum*
		*Exiguobacterium artemiae/sibiricum*
		*Exiguobacterium mexicanum*
		*Exiguobacterium oxidotolerans*
		*Exiguobacterium soli*
		*Exiguobacterium* sp.
	*Glutamicibacter*	*Glutamicibacter* spp.
	*Kocuria*	*Kocuria carniphila*
	*Luteococcus*	*Luteococcus japonicus*
	*Microbacterium*	*Microbacterium* sp.
	*Paenarthrobacter*	*Paenarthrobacter aurescens*
	*Pseudarthrobacter*	*Pseudarthrobacter chlorophenolicus*
	*Sanguibacter*	*Sanguibacter inulinus*
*Bacteroidetes*	*Chryseobacterium*	*Chryseobacterium indoltheticum*
	*Myroides*	*Myroides odoratimimus*
*Deinococcota*	*Deinococcus*	*Deinococcus budaensis/piscis*
*Pseudomonadota*	*Buttiauxella*	*Buttiauxella agrestis*
		*Buttiauxella MabrTax2*
*Verrucomicrobiota*	*Faecalibacterium*	*Faecalibacter rhinopitheci*

**Table 3:** Most common ^1^ culturable bacterial species in different stables.

Stable	Type	Dominant Species
A	Small private stable	*Bacillus cereus*
		*Exiguobacterium acetylicum*
		*Macrococcus carouselicus*
		*Staphylococcus aureus*
B	Large riding school	*Bacillus cereus*
		*Carnobacterium inhibens*
		*Exiguobacterium artemiae/sibiricum*
		*Macrococcus carouselicus*
C	Large riding school	*Bacillus cereus*
		*Bacillus pumilus*
		*Carnobacterium inhibens*
		*Exiguobacterium mexicanum*
		*Macrococcus carouselicus*
		*Pantoea agglomerans*
		*Streptococcus parauberis*
D	Large riding school	*Bacillus cereus*
		*Carnobacterium inhibens*
		*Staphylococcus equorum*

^1^ Bacterial species present in at least three horses within a stable, i.e., three out of four horses in stables B and C, and all three horses in stables A and D.

## Data Availability

The raw data supporting the conclusions of this article will be made available by the authors on request.
